# Executable explanation traces for legal LLM predictions via retrieval-augmented codification

**DOI:** 10.3389/frai.2026.1905145

**Published:** 2026-08-20

**Authors:** Haoyang Chen, Kumiko Tanaka-Ishii

**Affiliations:** Department of Computer Science and Communications Engineering, School of Fundamental Science and Engineering, Waseda University, Tokyo, Japan

**Keywords:** executable explanations, explainable artificial intelligence, large language models, legal artificial intelligence, legal judgment prediction, program-aided reasoning, retrieval-augmented generation, source support

## Abstract

Explainability is particularly challenging in legal prediction, where decisions are expected not only to be accurate but also to be justified under explicit legal norms and open to external scrutiny. Legal reasoning therefore provides one of the most demanding settings for explainable AI because a legal determination often depends on information distributed across the input record, external legal sources, procedural context, evidentiary assumptions, and institutional judgment. Direct outcome generation hides this decision path, while natural-language rationales and retrieved citations do not necessarily show whether a legal condition was actually applied. This article presents studies of retrieval-augmented codification as a way to produce executable explanation traces for legal large language model (LLM) predictions. The goal is not to generate human-facing rationales directly, but to produce auditable artifacts: short programs that extract signals, apply program-level conditions, follow branches, and produce final labels under a fixed output schema. The approach retrieves legal sources and fold-safe in-domain examples, compiles them into an executable intermediate representation, and refines the program using model feedback. The resulting trace records retrieved materials, generated conditions, feedback, and the final prediction. We evaluate this framework on five legal benchmarks covering Chinese criminal law, European human-rights cases, U.S. class-action complaints, Indian judgments, and Japanese tort cases. The evaluation asks not only whether the predicted label is correct, but also whether the explanation is executable, whether its conditions are supported by retrieved legal sources, whether it responds to legally material edits, and where it fails. The results show that executable explanations can be obtained without sacrificing predictive utility: the full codification setting improves mean label accuracy over direct prompting, chain-of-thought prompting, one-pass codification, and text-based retrieval baselines. More importantly, trace analysis reveals why legal explainability is difficult. Codification is most informative when decisions depend on definitions, thresholds, exceptions, and other rule-like components. It is less complete when benchmark labels depend on procedural, evidentiary, or discretionary factors outside the input record. These findings position retrieval-augmented codification as a practical explainable artificial intelligence (XAI) tool for studying when legal LLM predictions can be explained through explicit, source-supported decision paths.

## 1 Introduction

Explainability is particularly challenging in legal prediction, where decisions are expected not only to be accurate but also to be justified under explicit legal norms and open to external scrutiny. Legal reasoning therefore provides one of the most demanding settings for explainable AI because a legal determination often depends on information distributed across the input record, external legal sources, procedural context, evidentiary assumptions, and institutional judgment. Direct outcome generation hides this decision path, while natural-language rationales and retrieved citations do not necessarily show whether a legal condition was actually applied. This motivates an auditability-oriented view of legal explainable artificial intelligence (XAI): instead of asking only whether a plausible rationale accompanies an answer, we ask whether the reasoning path can be inspected, executed, and diagnosed.

Legal judgment prediction provides a useful testbed for this problem. It is commonly formulated as a text-to-label task, including charge prediction, article prediction, court outcome prediction, and liability prediction (Xiao et al., 2018; Chalkidis et al., 2019; Semo et al., 2022; Malik et al., 2021; Yamada et al., 2025). Given a case description, a model predicts a charge, violated article, court outcome, or liability label. This formulation is useful for benchmark evaluation because it gives a clear target for comparison. However, when the task is solved by direct large language model (LLM) label generation, the decision path is largely hidden. A correct label may result from applying a legal definition, recognizing a statutory exception, matching a similar case, or exploiting a dataset-specific correlation. The final label alone does not show which conditions were used, whether they were supported by retrieved legal sources, or where the prediction would fail under a legally material change.

This difficulty is not solved by natural-language rationales alone. Chain-of-thought prompting asks the model to produce intermediate reasoning text (Wei et al., 2022), and retrieval-augmented prompting can provide legal materials as context (Lewis et al., 2020; Gao et al., 2024). These methods can make an answer more readable and better informed. However, a rationale may mention a rule without making that rule determine the output, and a retrieved passage may appear in the prompt without being operationally used in the final prediction. This creates a gap between explanation as a textual justification and explanation as an applied decision process.

This article presents studies of retrieval-augmented codification as a way to produce executable explanations for legal LLM predictions. By codification, we mean using executable programs as intermediate representations for tasks originally expressed in natural language. Instead of asking a model to output a legal label directly, codification asks it to produce a program that extracts relevant signals, applies explicit program-level conditions, follows branches, and computes the label. The generated program is not treated as the law itself, nor as a complete human-facing legal explanation. Its value is audit-oriented and diagnostic: it turns direct label generation into an inspectable trace whose conditions, branches, retrieved sources, and failures can be examined.

The technical basis of this study is retrieval-augmented codification, a framework introduced in our previous work for converting natural-language tasks into executable intermediate representations through retrieval and iterative refinement (Chen and Tanaka-Ishii, 2026). Building on that framework, this study focuses on its role in legal AI: we ask whether the codified representation can be used not only to obtain a prediction, but also to expose an auditable text-to-label path in legal benchmark tasks. The novelty of the present study therefore lies in the legal instantiation and evaluation of retrieval-augmented codification as an executable trace: legal-source retrieval, fold-safe use of in-domain examples, trace-level diagnostics, material-edit sensitivity, and failure analysis across legal benchmarks. This shifts the emphasis from codification as a general reasoning procedure to codification as an auditability-oriented mechanism for legal LLM prediction.

Legal prediction is a hard setting for executable explanation because many relevant conditions are not contained in the input text. A criminal charge may depend on statutory definitions and thresholds. A human-rights article prediction may depend on doctrinal conditions. A civil or class-action outcome may depend on procedural requirements, defenses, evidentiary posture, or judicial discretion. The model must therefore connect the case text to external legal materials. Retrieval supplies legal sources and in-domain examples, while codification attempts to turn them into explicit executable conditions. Iterative refinement then revises the program using model feedback when the output schema is violated, when the program fails to produce a valid prediction, or when additional legal material is needed to implement a missing condition.

We use legal judgment prediction benchmarks as a cross-jurisdictional testbed for three questions. First, why is legal prediction difficult to explain with direct labels or free-form rationales? Second, when does retrieval-augmented codification produce useful executable explanations? Third, what failure modes become visible when the explanation trace is inspected? To answer these questions, we evaluate on five legal benchmarks: Chinese AI and Law (CAIL), European Convention on Human Rights (ECHR), Caselaw Access Project (CAP), Indian Legal Documents Corpus (ILDC), and Japanese Tort Dataset (JTD) (Xiao et al., 2018; Chalkidis et al., 2019; Semo et al., 2022; Malik et al., 2021; Yamada et al., 2025). These datasets cover Chinese criminal law, European human-rights cases, U.S. class-action complaints, Indian judgments, and Japanese tort cases, and therefore differ in language, jurisdiction, legal domain, and label structure.

Our evaluation treats label accuracy as a utility check rather than the primary endpoint. A method that produces explanations but collapses in predictive performance would be difficult to interpret as a useful legal AI framework. We therefore compare direct prompting, chain-of-thought prompting, retrieval baselines, one-pass codification, iterative retrieval, and retrieval-augmented codification. Beyond label accuracy, we analyze explanation-level properties: executability of the final program, refinement depth, final-program exceptions, source support for program-level conditions, sensitivity to legally material edits, and representative failure modes.

The results support three observations. First, executable explanations are most informative when the relevant part of the legal decision can be expressed as explicit conditions over facts and retrieved legal sources. Definitions, thresholds, temporal requirements, exceptions, and statutory elements often fit this form. Second, the legal source and example source play different explanatory roles. Retrieved legal sources supply normative content, while in-domain examples and code-like exemplars help construct task-specific mappings and stable program structure. Third, explanation failures reveal limits that are hidden by label accuracy alone. Some failures are local and repairable, such as missing exceptions or miscompiled thresholds. Others reflect a deeper mismatch between the benchmark label and what can be recovered from the input text and retrieved legal sources, such as outcome underdetermination caused by procedural, evidentiary, or discretionary factors outside the record.

Existing explainable AI methods typically seek human-understandable rationales. In contrast, our objective is different. We aim to produce executable reasoning traces that can be inspected, verified, and analyzed computationally. The resulting artifacts are not necessarily intended to be directly interpretable by legal practitioners. Rather, they support auditability: the ability to identify unsupported claims, missing evidence, retrieval failures, and reasoning inconsistencies through executable analysis.

This study makes the following contributions.

- We instantiate retrieval-augmented codification for legal LLM prediction and study it as an auditability-oriented trace framework: the output is not only a label, but an executable explanation trace that can be inspected computationally.
- We analyze why legal prediction is a difficult setting for executable trace-based explanation, distinguishing rule-like conditions from procedural, evidentiary, and discretionary factors outside the input record.
- We evaluate the framework across five legal benchmarks from different jurisdictions and legal domains, using label accuracy as a utility check and trace-level metrics as audit diagnostics.
- We study trace-level behavior, including executability, refinement depth, final-program exceptions, source support, material-edit sensitivity, and representative failure modes.
- We identify when executable traces are informative and when they break down, separating errors caused by missing legal sources, incorrect compilation, unsupported conditions, label–concept mismatch, and information absent from the input record.

The rest of the study is organized as follows. Section 2 positions the study relative to explainable AI, legal prediction, retrieval-augmented legal natural language processing (NLP), executable intermediates, and rules-as-code. Section 3 describes retrieval-augmented codification, the executable trace, as well as the datasets, baselines, knowledge sources, and metrics. Section 4 reports predictive utility and explanation-level results. Section 5 presents qualitative case studies. Section 6 discusses what executable explanations reveal and what they do not establish, and Section 7 concludes.

## 2 Related study

### 2.1 Explainable AI for legal LLM prediction

Explainable AI studies how automated decisions can be made understandable to users, developers, and affected stakeholders. This problem is especially important in high-stakes domains because black-box predictions can undermine trust, error diagnosis, and contestability (Das and Rad, 2020). Legal prediction is a particularly demanding setting for XAI. A legal label may depend not only on the input text, but also on external legal sources, institutional procedure, evidentiary assumptions, and discretionary judgment. An explanation must therefore do more than restate the predicted label; it should help identify which conditions were applied and whether relevant sources support those conditions.

This concern is also reflected in recent study in *Frontiers in Artificial Intelligence*, especially its Technology and Law section, where legal AI is studied in relation to judicial decision tools, legal concept alignment, algorithmic accountability, legal document summarization, and AI governance. For example, recent studies examine AI decision tools in court settings and audit-as-code approaches for AI assurance (Rodríguez-Salcedo et al., 2025; Muhammad et al., 2026). These studies show that legal AI evaluation is not only a question of predictive performance, but also a question of reviewability, uncertainty, and the relation between AI outputs and legal sources.

Existing studies on legal and high-stakes XAI emphasize that explanations should support scrutiny, actionability, and contestability rather than only readability (Mansi et al., 2025). Argumentation-based approaches to legal AI make a related point: legal reasoning is often defeasible, contestable, and value-sensitive, so explanations should expose premises, conflicts, and inferential structure (Prajescu and Confalonieri, 2025). Our study follows *the same broad motivation, but studies a different explanation object. Instead of producing a free-form rationale or an argument graph, we produce an executable trace: a program-like artifact that records retrieved sources, program-level conditions, branches, model feedback, and the final prediction.

This perspective is also motivated by limitations of *post-hoc* explanations. Natural-language rationales can be plausible without being faithful to the actual decision process, and retrieved citations can appear in a prompt without affecting the final answer. More generally, XAI methods themselves must be evaluated for reliability, because explanations can be incomplete, misleading, or vulnerable to manipulation (Baniecki and Biecek, 2023). We therefore evaluate explanations not only by whether they are readable, but by whether they are executable, source-supported, sensitive to legally material edits, and diagnostically useful when they fail.

### 2.2 Legal prediction and retrieval-augmented legal NLP

Legal judgment prediction provides a standard way to evaluate legal NLP systems as text-to-label models. It has been studied as a central task in legal NLP, together with broader legal language understanding and legal reasoning benchmarks (Cui et al., 2022; Chalkidis et al., 2022; Guha et al., 2023). Existing benchmarks cover different legal systems and label schemes, including criminal charge prediction, human-rights article prediction, class-action outcome prediction, court judgment prediction, and tort judgment prediction (Xiao et al., 2018; Chalkidis et al., 2019; Semo et al., 2022; Malik et al., 2021; Yamada et al., 2025). These datasets make legal reasoning tasks measurable at scale. At the same time, the label in such benchmarks often compresses several layers of legal decision-making. A charge, violated article, or outcome label may depend on statutory elements, factual characterization, evidence, procedural posture, and institutional practice. Accuracy therefore shows whether a system matches the benchmark label, but not which legal conditions were applied or whether the prediction is supported by retrieved legal sources.

Retrieval-augmented generation (RAG) addresses one part of this problem by conditioning generation on external documents (Lewis et al., 2020; Gao et al., 2024). In legal tasks, retrieval quality is especially important because the relevant rule may not be fully stated in the case text, and unreliable retrieval can directly affect the generated legal answer (Reuter et al., 2025). Iterative retrieval methods further show that retrieval can be interleaved with intermediate reasoning steps rather than used only once before answer generation (Trivedi et al., 2023). This is useful for multi-step legal problems where the missing legal source may only become clear after an initial interpretation of the facts.

Recent legal RAG studies make this point more concrete. Athena applies retrieval-augmented generation to legal judgment prediction on CAIL2018, using an accusation-oriented knowledge base, query rewriting, and retrieval analysis to improve prediction performance (Peng and Chen, 2024). NyayaRAG studies a more realistic Indian common-law setting by augmenting factual case descriptions with statutory provisions and semantically retrieved prior cases, evaluating both decision prediction and legal explanation quality (Nigam et al., 2025). These studies show that retrieval is useful for legal prediction when the relevant legal knowledge is outside the input text.

Our study addresses a later step in the explanation pipeline. Once legal knowledge is retrieved, we ask whether it becomes an explicit condition in the decision procedure. We therefore use retrieval as the source of legal material, but evaluate the codification step that turns retrieved material into executable conditions and branches. This separates our setting from legal RAG systems that use retrieved materials primarily as prompt context for answer generation.

### 2.3 Executable intermediates and executable explanations

A related line of study uses executable programs as intermediate reasoning objects. Program-aided language models generate code to perform computations that are difficult to carry out reliably in natural language (Gao et al., 2023). Program-of-thought prompting separates computation from natural-language reasoning by delegating numerical or symbolic operations to a program interpreter (Chen et al., 2023). Chain-of-Code extends this direction by combining code execution with LMulator-style simulation for natural-language tasks whose steps are not always directly executable (Li et al., 2024). More broadly, ReAct-style methods interleave reasoning with actions that query external sources or environments (Yao et al., 2023), and CodeAct uses executable Python code as a unified action interface for LLM agents (Wang et al., 2024).

These studies show that code can serve as more than a final product. It can function as an intermediate representation that makes part of the reasoning process explicit and testable. In the present study, we treat this property as an explanation mechanism: a natural-language legal task is transformed into an executable trace whose variables, program-level conditions, and branches expose how the answer is produced. This is useful for legal prediction because legal labels often depend on conditions that can be stated as elements, exceptions, thresholds, or temporal requirements.

Legal codification also introduces a difficulty that is less central in many arithmetic, symbolic, or agent-control tasks. The input text usually contains facts, while the applicable legal rules may reside outside the input. A program generated only from the case description may therefore encode the wrong rule, omit an exception, or rely on model memory. This motivates retrieval-augmented codification: retrieved legal sources supply the legal content, while the executable program exposes how that content is applied. Our study follows the general idea of executable intermediates, but studies it in a legal XAI setting where external sources and benchmark labels must be connected through explicit decision conditions.

### 2.4 Executable legal rules and limits of legal formalization

Executable representations have a long history in AI and Law. Rules-as-code and computational-law approaches aim to represent legal norms in operational forms that can be inspected, tested, maintained, and consumed by software systems (Waddington, 2021; Mohun and Roberts, 2020; Mérigoux et al., 2021). Catala is a representative example of this direction: it is designed as a programming language for translating statutory law into executable implementations while making the structure of the legal text explicit (Mérigoux et al., 2021). Statutory reasoning benchmarks also show that correctness often depends on explicit legal definitions, exceptions, and rule interactions rather than surface similarity alone (Holzenberger et al., 2020). A related study on legal debugging and rule revision treats mismatches between rule implementations and intended legal behavior as objects that can be tested and repaired (Fungwacharakorn and Satoh, 2022).

Our setting is related but different. We do not construct an authoritative executable legal codebase, nor do we claim that generated programs are legal norms. Instead, we use generated executable traces as empirical artifacts for explaining and diagnosing benchmark predictions. A trace records the retrieved legal sources, generated program-level conditions, model feedback, and final prediction. This makes it possible to ask whether a prediction was produced through an explicit condition, whether the condition was supported by retrieved legal sources, and where the explanation failed.

This perspective also connects to robustness and limits of legal formalization. Legal knowledge sources can be incomplete, outdated, jurisdictionally mismatched, or misleading. In a text-only system, such problems may be hidden inside the final answer. In an executable trace, they can appear as missing conditions, unsupported branches, schema failures, or predictions that depend on conditions absent from the retrieved legal sources. Executability therefore serves as an observability condition: it does not establish legal correctness, but it provides a concrete explanation object for studying how retrieved knowledge affects the decision path. This view is close to recent audit-as-code and policy-as-code studies discussed in *Frontiers in Artificial Intelligence*, where executable representations are used to support AI assurance and review rather than to replace human judgment (Muhammad et al., 2026).

## 3 Materials and methods

### 3.1 Problem setting: legal prediction as explanation

We consider a legal judgment prediction dataset


$$\begin{array}{l} D={\left\{\left(t_{i},a_{i}\right)\right\}}_{i=1}^{\left|D\right|}, \end{array}$$ (1)

where *t*_*i*_ is a legal input text, such as a case description or complaint, and *a*_*i*_ is the benchmark label. The label may be a criminal charge, a violated article, a binary outcome, or a liability judgment, depending on the dataset. Standard prompting methods map the input text directly to a prediction â. In this study, we study a different output object: an executable explanation trace. The model first produces a program-like intermediate representation *c*, and the prediction â is obtained from the final codified representation. The explanation is therefore not a separate *post-hoc* text; it is the program-level path that produces the label.

The program is required to expose the intermediate signals used for prediction. Concretely, it extracts or defines legally relevant variables, applies explicit program-level conditions, and outputs the final prediction under a fixed machine-readable schema. We call such program-level conditions *predicates* when they can affect the final prediction. A predicate may test a factual signal, a legal element, an exception, a threshold, a temporal requirement, or a procedural condition. The term is used operationally in this study: a predicate is a condition used inside the generated decision procedure, not a complete formal representation of a legal norm.

For example, in a criminal-law charge prediction task, a codified program may extract an amount, check whether the act involved unlawful taking, and then test a condition such as amount_taken >= theft_threshold. This threshold check is a predicate in our sense because its truth value can change the predicted charge. These predicates are the units we inspect in the explanation trace: we can ask whether they were used, whether they affected a branch, and whether they were supported by retrieved legal sources.

### 3.2 Framework overview

**Figure 1:**
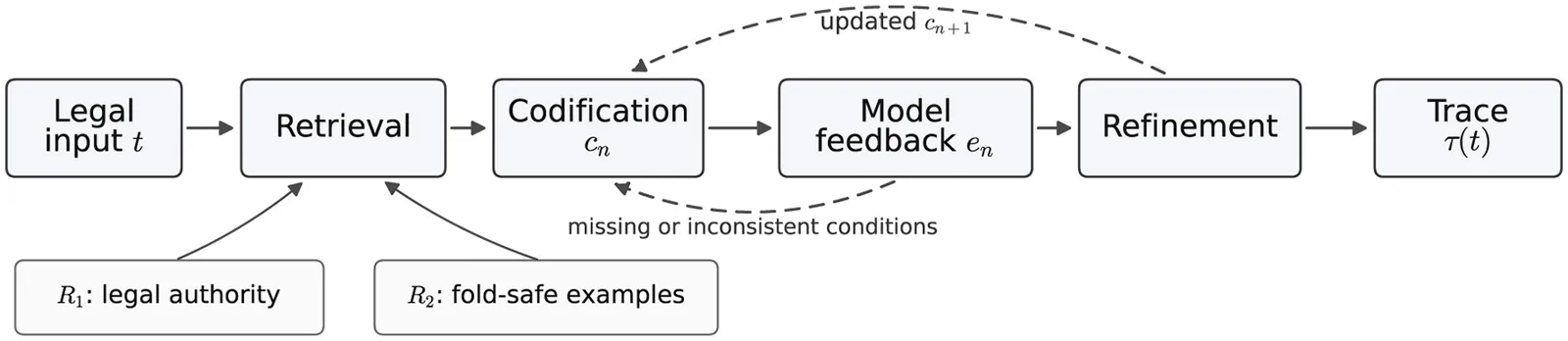
Retrieval-augmented codification framework for executable explanation. Given a legal input *t*, the system retrieves legal authority from *R*_1_ and fold-safe in-domain examples from *R*_2_, represents the decision path as a program-like intermediate object *c*_*n*_, obtains model feedback *e*_*n*_, and refines the program when the feedback indicates missing or inconsistent conditions. The final output is an executable trace τ(*t*), rather than only a predicted label.

Figure 1 illustrates the full framework. Given a legal input *t*, the system retrieves legal materials, generates an initial program *c*_0_, obtains model feedback on the program, and iteratively revises the program until it produces a valid output or reaches the iteration cap. The final artifact is an executable explanation trace rather than only a label: it records the retrieved materials, codified predicates, model feedback, and final prediction.

The technical basis of this study is retrieval-augmented codification, introduced in our previous work as a general framework for converting natural-language tasks into executable intermediate representations through retrieval and iterative refinement. In this study, we use the framework for a different purpose: to study explainability in legal LLM prediction. The trace is therefore evaluated not only as an intermediate computation, but as an explanation object that exposes which legal conditions were applied, which sources were available, and where the explanation fails.

The framework has four components. First, retrieval supplies external legal materials and in-domain examples that may be absent from the input text. Second, codification translates the input and retrieved materials into a short Python program that maps legal signals to a prediction. Third, iterative refinement uses model feedback to revise the program when the output schema is violated, when required information is missing, or when additional legal material is needed. Fourth, trace construction records the sequence of programs, retrieved materials, and feedback so that the decision path can be inspected.

This framework is retrieval-augmented because the legal content used by the program is supplied through *R*_1_ and *R*_2_, described in Section 3.3. It is iterative because the codified representation is not fixed after the first generation; it can be revised through model feedback, as described in Section 3.4. These two parts serve different explanatory roles: retrieval supplies what legal or task-specific information may be needed. At the same time, iteration tests whether the current codified representation is complete enough to produce a valid prediction.

### 3.3 Knowledge sources for explanation

The retrieval component uses two knowledge sources. The first source, *R*_1_, contains legal authority for the corresponding jurisdiction and task. Depending on the dataset, this includes statutes, legal articles, regulations, judicial materials, or other curated legal texts. *R*_1_ is intended to supply legal content that may be absent from the input text, such as definitions, thresholds, exceptions, and doctrinal conditions.

The second source, *R*_2_, contains in-domain solved examples. These examples provide task-specific mappings between fact patterns and benchmark labels. In some settings, *R*_2_ is represented as natural-language examples; in others, solved examples are converted into short code-like snippets. To avoid direct test leakage, *R*_2_ is constructed fold-safely. For each evaluation fold, only training instances are available for retrieval, and the held-out instance is excluded from the retrieval pool.

The two sources play different explanatory roles. *R*_1_ provides the legal content that can support program-level conditions. *R*_2_ provides examples of how similar benchmark instances are mapped to labels. The full codification setting uses both sources, while ablation variants remove or change one of them to test how different knowledge forms affect both prediction and explanation.

### 3.4 Iterative codification and explanation trace construction

Given an input *t* and retrieved materials *r*_*n*_, the model generates or revises a program-like representation *c*_*n*_. The program is written so that legally relevant facts, conditions, exceptions, and label decisions are expressed as explicit variables, predicates, and branches. During refinement, the system may use LMulator-style model feedback, following Chain-of-Code (Li et al., 2024), to diagnose underspecified or inconsistent intermediate representations. This feedback is used to revise the program, not to produce the final label directly.

The refinement process produces a sequence


$$\begin{array}{l} c_{0}\to c_{1}\to \cdots \to c_{N}=c^{⋆}, \end{array}$$ (2)

where *c*^⋆^ is the final program used to produce the prediction â. After refinement stops, â is obtained by executing *c*^⋆^ under the required output schema. Thus, LMulator-style feedback affects the construction of *c*^⋆^, whereas executability and exception rate are measured on the actual execution of *c*^⋆^. Refinement is triggered when the current representation fails to produce a valid output, violates the required schema, or indicates that additional legal authority is needed. The model may then revise the program and optionally issue a new retrieval query. The process stops when a valid output is produced, when no further retrieval query is produced, or when a maximum number of iterations is reached.

For each input *t*, the system records the explanation trace


$$\begin{array}{l} \tau \left(t\right)=\left(c_{0},r_{0},e_{0};c_{1},r_{1},e_{1};\ldots ;c_{N},r_{N},e_{N}\right), \end{array}$$ (3)

where *c*_*n*_ is the codified representation at iteration *n*, *r*_*n*_ is the retrieved material available at that iteration, and *e*_*n*_ is model feedback. The trace is used for explanation analysis in three ways. First, it shows whether an explicit decision procedure produced the final prediction. Second, it shows which retrieved materials were available when the program was generated or revised. Third, it exposes the predicates and branches that affected the prediction. These properties allow us to separate failure modes that are indistinguishable from accuracy alone, such as retrieval failure, incorrect compilation, schema failure, unsupported predicate, and label–concept mismatch.

### 3.5 Scope of the executable trace

The generated program is an explanation artifact, not an authoritative legal rule. Its value comes from making part of the decision path observable: variables, predicates, branches, retrieved legal sources, and outputs can be inspected after generation. This makes it possible to ask whether a prediction depended on a stated condition, whether that condition was supported by retrieved legal sources, and where the explanation failed.

This explanation is limited. A trace may run successfully while omitting an exception, applying an unsupported condition, or computing a proxy for the benchmark label. Thus, executability does not establish legal correctness, and a generated trace should not be read as a complete formalization of the law. Its role in this study is narrower: it exposes which parts of a legal benchmark decision can be represented as explicit conditions, and where this representation breaks down.

### 3.6 Experimental setup

The experiments are designed to evaluate retrieval-augmented codification as an explainable-AI mechanism for legal prediction. We ask four questions. First, can executable explanations be produced without sacrificing label accuracy? Second, are the final explanation traces executable and stable under refinement? Third, are the program-level conditions in the explanations supported by retrieved legal sources? Fourth, what failure modes become visible when the explanation trace is inspected? We therefore report both predictive utility and explanation-level properties, including refinement rounds, executability of the final program *c*^⋆^, exception rate of the final program, source support, material-edit sensitivity, and qualitative failure categories.

### 3.7 Datasets

We evaluate on five legal judgment benchmarks covering different jurisdictions, languages, and label schemes. Table 1 summarizes the datasets and the legal-source corpus used for retrieval. The datasets include both element-like classification tasks, where labels are closely tied to statutory or doctrinal conditions, and outcome-oriented tasks, where labels may also depend on procedure, evidence, or institutional judgment. This makes them useful as a testbed for legal XAI. Some labels should be explainable through explicit legal conditions, while others reveal the limits of explanation from the input record alone.

**Table 1:** Legal datasets and legal-source corpora used in the evaluation.

Dataset	Jurisdiction	|D|	|*A*|	Legal-source corpus *R*_1_
CAIL	China	50,543	150	Criminal law/judicial interpretations
CAP	U.S.	3,000	2	U.S. Code
ECHR	Europe	4,362	20	HUDOC articles/ECHR materials
ILDC	India	1,507	2	Indian Supreme Court materials
JTD	Japan	6,508	2	Japanese Civil Code

CAIL is a Chinese criminal-law benchmark for charge prediction (Xiao et al., 2018). The model predicts one or more criminal charges from a case fact description. CAP is a U.S. class-action prediction benchmark where the task is to predict a binary legal outcome from class-action complaint texts (Semo et al., 2022). ECHR is a European human-rights benchmark in which the model predicts violated articles from case descriptions (Chalkidis et al., 2019). ILDC is an Indian Supreme Court case prediction dataset (Malik et al., 2021). JTD is a Japanese tort-case dataset for legal judgment prediction with rationales (Yamada et al., 2025). These datasets differ in jurisdiction, input style, label space, and the degree to which the label can be expressed as explicit legal conditions.

For each dataset, we construct or use a corresponding legal-source corpus *R*_1_. The legal-source corpus is intended to supply legal materials that may be absent from the input text, such as statutory definitions, article conditions, procedural requirements, and exceptions. The exact corpus differs by dataset: Chinese criminal law and judicial interpretations for CAIL, U.S. legal materials for CAP, HUDOC and ECHR-related materials for ECHR, Indian Supreme Court materials and case-law context for ILDC, and the Japanese Civil Code for JTD.

### 3.8 Baselines and variants

We compare retrieval-augmented codification with prompting, retrieval, and code-based baselines. The compared methods are designed to isolate four factors: whether the method uses external knowledge, whether it uses a code-like intermediate representation, whether it performs iterative retrieval/refinement, and whether the final answer is obtained through an executable trace.

Table 2 summarizes the methods. Direct prompting maps the input text *t* directly to a predicted label. CoT adds a minimal chain-of-thought instruction (Wei et al., 2022). CoC is a one-pass codification baseline based on Chain-of-Code (Li et al., 2024): the model generates a code-like representation from the input and obtains the answer through the code execution/emulation procedure, but does not use retrieval-driven refinement.

**Table 2:** Baselines and codification variants for an input *t*.

Method	Retrieval	System	Acquiring â
Direct	–	LLM(*t*)	Text
CoT	–	LLM(*t* + “step by step”)	Text
CoC	–	CoC(*t*)	Execute *c*_0_
RAG_NL_	*R*_1_ + (*d* ∈ *R*_2_)	LLM(*t*⊕retrieved)	Text
RAG_CODE_	*R*_1_ + (*s* ∈ *R*_2_)	LLM(*t*⊕retrieved)	Text
IRCoT	*R*_1_ + (*d* ∈ *R*_2_)	Interleaved retrieval + CoT	Text
Ours_NL_	*R*_1_ + (*d* ∈ *R*_2_)	Iterative codification	Execute *c*^⋆^
Ours_1PASS_	*R*_1_ + *R*_2_	One-pass codification	Execute *c*_0_
Ours (Full)	*R*_1_ + *R*_2_	Iterative codification	Execute *c*^⋆^

*d*/*s* indicate whether the *R*_2_ exemplar pool is provided as text or code.

The retrieval baselines use external knowledge but do not require the retrieved material to be compiled into an executable explanation. RAG_NL_ retrieves legal authority and in-domain textual examples, then asks the model to produce a textual answer. RAG_CODE_ retrieves legal authority and code-like exemplars, but still produces the final answer as text. IRCoT interleaves retrieval with chain-of-thought sentence generation: intermediate CoT steps guide subsequent retrieval, and the retrieved evidence is then used to continue the reasoning process (Trivedi et al., 2023). These baselines test whether retrieval and iterative evidence gathering are sufficient without codifying the decision path.

The final variants produce executable explanation traces. The text-example codification variant uses legal authority and in-domain textual examples, then obtains the final prediction through iterative codification. We denote this variant as Ours_NL_. The full codification setting uses both legal authority and the full in-domain example pool, including code-like exemplars. We denote this variant as Ours (Full).

To isolate the effect of refinement, we also include a no-refinement ablation, Ours_1PASS_. This variant uses the same initial *R*_1_ and *R*_2_ retrieval results as Ours (Full), but stops after the first generated program *c*_0_. The prediction is obtained by executing *c*_0_, with no model feedback, program revision, or subsequent retrieval. This ablation separates the effect of the initial retrieval-conditioned codification step from the additional effect of refinement.

### 3.9 Experimental knowledge sources

The retrieval component uses two knowledge sources, *R*_1_ and *R*_2_. *R*_1_ is the legal-source corpus associated with each dataset. It provides legal materials that may be absent from the input text, such as statutory definitions, article conditions, thresholds, exceptions, and jurisdiction-specific rules. As shown in Table 1, *R*_1_ is constructed from the matching jurisdiction for each benchmark.

*R*_2_ is an in-domain solved-example corpus. It is used to provide task-specific mappings between fact patterns and labels. We use two forms of *R*_2_. In the natural-language form, each retrieved item is a solved example represented as text. In the code-like form, each solved example is converted into a short snippet that represents the mapping from relevant facts to the benchmark label. The purpose of *R*_2_ is not to retrieve the answer to the current test instance, but to provide analogical structure for how similar benchmark instances are codified.

To avoid direct test leakage, *R*_2_ is constructed fold-safely. We use *k*-fold evaluation. For each fold, the held-out portion is used for evaluation, and only the remaining *k* − 1 folds are indexed as the in-domain example pool. Thus, the current test instance is not available for retrieval as a solved example. Unless otherwise stated, we use *k* = 5.

The distinction between *R*_1_ and *R*_2_ is important for interpreting both predictions and explanations. *R*_1_ supplies legal content: definitions, elements, exceptions, and jurisdiction-specific authority. *R*_2_ supplies task-specific patterns: how benchmark inputs are usually mapped to labels. A method can benefit from one source while failing to use the other. The baselines in Table 2 are designed to separate these roles.

### 3.10 Evaluation metrics

We report both label-level and explanation-level metrics—the label-level metric measures predictive utility: whether the method predicts the benchmark label. The explanation-level metrics measure how the codified explanation behaves.

#### 3.10.1 Accuracy

Accuracy is used as a predictive-utility metric:


$$\begin{array}{l} \text{Acc}=\frac{1}{\left|D\right|}\overset{\left|D\right|}{\underset{i=1}{\sum }}1\left\{{â}_{i}=a_{i}\right\}. \end{array}$$ (4)

For binary and multi-class tasks, â_*i*_ is compared with the benchmark label under the dataset's label schema. For multi-label tasks, we use the strict matching rule defined for the corresponding benchmark. In this study, accuracy checks whether executable explanations are obtained without collapsing task performance; it is not the only endpoint of the evaluation.

Because the experiments are conducted under fold-safe evaluation, we report predictive accuracy as mean accuracy across evaluation folds. For Ours (Full), the proposed full codification setting, we additionally report the standard deviation across folds in the main results table. This fold-level uncertainty is used to indicate the stability of the proposed setting and to avoid over-interpreting small point-estimate differences. In the Section Results, differences whose magnitude is comparable to the reported fold-level variability are described as comparable rather than as clear improvements.

#### 3.10.2 Refinement rounds

For codified variants, we report the number of refinement rounds *N*. We report both the average and maximum *N* for the full retrieval-augmented codification setting. This metric indicates how many revisions are needed before the system obtains the final codified representation *c*^⋆^. A small *N* suggests that refinement mainly performs localized completion or repair, while a larger *N* may indicate that several conditions must be retrieved or reconciled.

#### 3.10.3 Executability

Executability is the fraction of instances for which the final program *c*^⋆^ runs successfully and produces the required output format. This metric concerns the final codified representation, not whether every intermediate representation during refinement is executable. For explanation analysis, executability measures whether the explanation can be operationalized as a runnable and parseable decision procedure. It does not imply that the procedure is legally correct.

#### 3.10.4 Exception rate

Exception rate is the fraction of instances for which executing the final program *c*^⋆^ raises an exception. It is not the complement of executability, because a final program may also fail to produce a valid prediction through schema violation, missing output, unparsable output, or invalid label. Exception rate measures one concrete form of explanation failure.

#### 3.10.5 Trace-level diagnosis

For qualitative analysis, we inspect representative traces and group failures into recurring categories. These include missing retrieval, incorrect compilation of legal sources into predicates, schema failure or final-program exception, unsupported predicates, label–concept mismatch, and outcomes that depend on information absent from the input record. These categories are used to explain why a method succeeds or fails beyond the final accuracy value.

### 3.11 Implementation details

All methods use the same base language model, decoding settings, retrieval top-*k*, and context budget whenever applicable. The iterative codification variants additionally allow refinement up to *N*_max_ = 10. We therefore report the observed average/maximum refinement depth *N*, and include Ours_1PASS_ as a no-refinement ablation to separate the effect of the first retrieval-conditioned codification pass from later refinement. In the main experiments, we use GPT-5-chat as the base model. Across methods, we keep the retrieval setting, context budget, maximum output length, and decoding configuration fixed whenever they are applicable. The maximum number of refinement rounds is also fixed for the codified variants; in the main experiments, we use *N*_max_ = 10. If the refinement cap is reached, the latest available program is used only when it produces the required output format; otherwise, the instance is counted as non-executable.

For retrieval-based methods, the same *R*_1_ legal-source corpus and fold-safe *R*_2_ example pool are used whenever the method requires that source. Retrieved items are inserted into the prompt according to the method type: as natural-language context for RAG_NL_ and IRCoT, as code-like exemplars for RAG_CODE_, and as materials to be compiled into a codified representation for the iterative codification variants.

For codified variants, the model is required to output a program that produces a final prediction under a fixed machine-readable schema. During intermediate refinement, the system may request LMulator-style diagnostic feedback, following Chain-of-Code (Li et al., 2024), when the current representation is underspecified, schema-inconsistent, or fails to produce a valid output. This feedback is used only to revise the program. The final answer â is obtained by executing the final program *c*^⋆^ under the required output schema. If *c*^⋆^ cannot be executed or does not produce a valid schema-compliant output, the instance is counted as non-executable.

Complete implementation settings, including model version, decoding parameters, retrieval top-k, maximum refinement rounds, output budget, and fold construction, are provided in the Supplementary material.

## 4 Results

### 4.1 Predictive utility of executable explanations

Table 3 reports the main results across the five legal benchmarks. The table compares prompting baselines, retrieval baselines, one-pass codification, interleaved retrieval with CoT, the no-refinement ablation Ours_1PASS_, and the iterative codification variants. The rightmost block reports trace-level statistics for the full codification setting, which are analyzed in Section 4.2. In this subsection, we focus on label accuracy.

**Table 3:** Predictive utility and explanation-level behavior on legal judgment benchmarks.

Dataset	Direct	CoT	CoC	RAG_NL_	RAG_CODE_	IRCoT	Ours_NL_	ours_1PASS_	ours (Full)	*N*	Executability	Exception rate
CAIL	0.538	0.542	0.664	0.686	0.658	0.610	0.674	0.704	**0.724** (0.0142)	1.06/2	94%	5.15%
ECHR	0.511	0.531	0.547	0.598	0.477	0.613	0.619	0.621	**0.642** (0.0224)	2.86/9	87%	12.20%
CAP	0.570	0.606	0.552	0.580	0.550	0.552	0.652	0.657	**0.683** (0.0411)	1.12/3	85%	14.11%
ILDC	0.671	0.714	0.590	0.722	0.709	0.733	0.747	0.747	**0.759** (0.0113)	1.65/4	91%	8.72%
JTD	0.554	0.571	0.438	0.673	0.612	0.718	0.733	0.727	**0.748** (0.0421)	2.10/6	95%	4.33%
Mean	0.569	0.593	0.558	0.652	0.601	0.645	0.685	0.691	**0.711**	–	–	–

Accuracy is reported for all methods as mean accuracy across evaluation folds. For Ours (Full), the proposed full codification setting, fold-level standard deviation is shown in parentheses. The right block reports explanation-level behavior of the full codification setting. *N* is the average/maximum number of refinement rounds. Executability is the fraction of instances for which the final program *c*^⋆^ runs successfully and produces the required output. Exception rate is the fraction of instances for which executing the final *c*^⋆^ raises an exception. Bold values indicate the best-performing result for the corresponding dataset and metric among the compared methods.

The main pattern is that Ours (Full) achieves the highest mean accuracy across the evaluated legal benchmarks. It reaches a mean accuracy of 0.711, compared with 0.569 for Direct, 0.593 for CoT, 0.558 for one-pass CoC, 0.652 for RAG_NL_, 0.645 for IRCoT, 0.685 for Ours_NL_, and 0.691 for Ours_1PASS_. This result matters for the study's claim because the executable trace is not obtained by sacrificing benchmark utility. The method produces an inspectable decision procedure while preserving, and in mean accuracy improving, predictive utility under the evaluation setting. Small numerical gaps should be interpreted conservatively in light of the fold-level variability reported for Ours (Full).

The comparison separates the roles of retrieval, codification, and refinement. RAG_NL_ improves over Direct on all five datasets, showing that external knowledge is useful for legal prediction. However, RAG_NL_ still produces the final answer as text, so the use of retrieved legal sources remains implicit. The text-example codification variant further improves the mean accuracy from 0.652 to 0.685, suggesting that converting retrieved textual examples into an executable decision path adds value beyond retrieval alone. Ours_1PASS_ then tests whether the initial retrieval-conditioned codification step is sufficient without feedback-driven revision. In contrast, Ours (Full) tests the additional effect of refinement on top of the same initial retrieval condition.

Ours_1PASS_ uses the same initial *R*_1_ and *R*_2_ retrieval results as Ours (Full), but obtains the prediction by executing only the first generated program *c*_0_. The comparison between CoC and Ours_1PASS_ measures the effect of adding retrieved legal and in-domain materials to one-pass codification. The comparison between Ours_1PASS_ and Ours (Full) measures the additional effect of feedback-driven refinement and subsequent retrieval. This ablation therefore addresses whether the full method gains only from additional rounds, or whether the initial retrieval-conditioned codification step already contributes predictive utility.

The effect of refinement is dataset-dependent. When the first generated program already captures the relevant legal conditions, Ours_1PASS_ is close to Ours (Full). When the label requires additional condition repair, label-interface correction, or further retrieved material, refinement contributes more. This comparison is therefore not intended to show that additional rounds uniformly improve accuracy, but to identify when the first executable trace is sufficient and when refinement is needed to complete or repair it.

The table also clarifies the role of code-like exemplars. RAG_CODE_ does not consistently improve over RAG_NL_; in the mean score, it is lower than the plain-text retrieval baseline. This indicates that code-like examples are not automatically useful when they are inserted as passive prompt context and the model still produces a textual answer. Their benefit appears in the codification setting, where retrieved code-like structure is used to shape an executable program and is coupled with execution and refinement. Thus, the result should not be read as evidence that code exemplars are universally helpful, but as evidence that their usefulness depends on whether they participate in executable trace construction.

The gain over one-pass codification is also important. CoC performs competitively on CAIL, but its mean accuracy is lower than Direct because legal tasks often require information not contained in the input text. This supports the motivation for retrieval-augmented codification: executable explanation requires not only a code-like representation, but also legal sources and in-domain examples that supply missing definitions, exceptions, and task-specific mappings.

Across datasets, the size of the gain differs. CAIL and JTD show large improvements over Direct, from 0.538 to 0.724 and from 0.554 to 0.748, respectively. These tasks contain many decisions that can be expressed through relatively explicit elements, charge distinctions, or liability conditions. CAP and ECHR also improve, but their lower executability and higher exception rates in Table 3 suggest that outcome-oriented labels and multi-condition article prediction create more explanation brittleness. ILDC has stronger retrieval and CoT baselines, but the full codification setting still gives the best result, indicating that codification remains useful even when natural-language retrieval already performs well.

The key conclusion from Table 3 is therefore not simply that one method has the highest accuracy. Rather, the table shows that executable explanations remain useful under label-based evaluation. This makes the explanation analysis in the following sections meaningful: the trace is attached to a method that performs well on the benchmark, while exposing additional information about refinement, executability, exceptions, source support, and failure modes.

### 4.2 Operational behavior of explanation traces

Accuracy reports whether the final label matches the benchmark, but it does not show whether the prediction was produced through a runnable explanation. We therefore inspect three explanation-level properties of the full codification setting: the number of refinement rounds *N*, executability of the final program *c*^⋆^, and exception rate of the final program *c*^⋆^. These metrics do not establish legal correctness. They show whether the system can operationalize the decision path and where the explanation becomes brittle.

The refinement depth is small across all datasets. As shown in the right block of Table 3, the average number of refinement rounds ranges from 1.06 on CAIL to 2.86 on ECHR. The maximum also remains bounded: CAIL reaches at most 2 rounds, CAP 3, ILDC 4, JTD 6, and ECHR 9. This pattern suggests that the refinement loop is mostly used for localized completion and repair rather than long-horizon search. In typical cases, the system adds or corrects a small number of conditions, such as a missing definition, an exception, a threshold, or a branch needed to reconcile the retrieved legal sources with the input facts.

Executability is high overall but varies by dataset. CAIL and JTD have the highest executability, at 94% and 95%, respectively. ILDC also remains high, at 91%. ECHR reaches 87%, while CAP is lower, at 85%. This difference is meaningful for XAI. Datasets whose labels are closer to explicit elements or relatively stable legal conditions tend to yield more executable explanations. Outcome-oriented tasks create more opportunities for incomplete extraction, unstable branching, or schema-level failure.

Exception rate captures a different aspect of the final executable explanation. It measures whether executing the final *c*^⋆^ raises an exception. This metric is related to, but not the complement of, executability: executability requires *c*^⋆^ to run successfully and produce the required output format, while non-executability may also result from schema violations, missing predictions, unparsable outputs, or invalid labels. For example, ECHR has an executability of 87% and an exception rate of 12.20%, while CAP has an executability of 85% and an exception rate of 14.11%. Thus, the exception rate measures one major source of explanation failure, rather than all failures.

The dataset-level differences also support the distinction between prediction and explanation behavior. ECHR has the deepest refinement, with an average of 2.86 rounds and a maximum of 9, which is consistent with its article-prediction setting where several conditions may need to be reconciled. CAP has the lowest executability and the highest exception rate in Table 3. Although its label is binary, complaint outcomes can depend on procedural posture, pleading sufficiency, and case-specific legal theories. The explanation metrics make this complexity visible in a way that accuracy alone does not. CAIL and JTD, by contrast, combine high executability with low exception rates, suggesting that their benchmark labels more often admit compact executable explanations under the available legal sources and examples.

These results clarify the role of executability as an explanation metric. A high executability rate means that the system often produces a final program that runs and outputs the required format, but it does not imply legal completeness. Together with exception rate and refinement depth, executability serves as diagnostic evidence: it shows where codification yields a runnable explanation and where the explanation becomes unstable.

### 4.3 Effect of external knowledge on explanations

**Figure 2:**
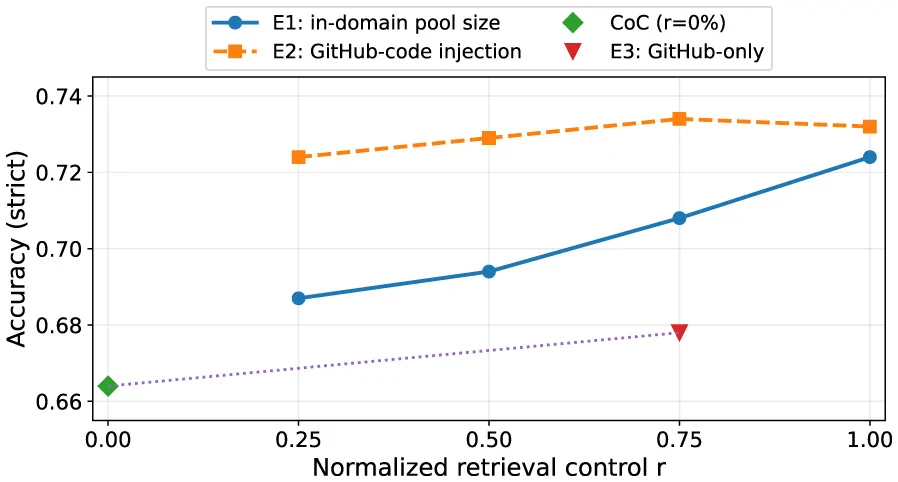
External-knowledge ablations on CAIL. The horizontal axis is a normalized control *r* ∈ [0%, 100%]. E1 varies the fraction *r* of the in-domain retrieval pool *R*_1_ + *R*_2_. E2 injects additional off-domain GitHub code into the retrieval corpus on top of E1. Discrete reference points show CoC at *r* = 0% and E3: GitHub-only at *r* = 75%.

We isolate the effect of external knowledge by changing only the retrieval corpus while keeping the LLM, prompts, and codification/refinement procedure fixed. We report this analysis on CAIL, the largest legal dataset in our evaluation, to obtain stable trends. Figure 2 summarizes three controlled settings. **E1** subsamples the in-domain retrieval pool *r*·(*R*_1_ + *R*_2_). **E2** augments E1 by injecting additional off-domain GitHub code snippets into the retrieval candidates; in-domain legal material remains available, and GitHub code is added as extra implementation scaffolding. **E3** uses a GitHub-only pool as the retrieval source.

#### 4.3.1 In-domain pool size

Increasing the fraction of the in-domain pool improves accuracy: 0.687 at 25%, 0.694 at 50%, 0.708 at 75%, and 0.724 at 100%. This monotonic trend supports the role of in-domain retrieval in executable explanation. The retrieved legal sources and solved examples supply definitions, thresholds, exceptions, and charge distinctions that can be represented as explicit predicates and branches in the final program.

#### 4.3.2 GitHub injection

Adding generic GitHub code on top of in-domain retrieval also helps, but in a different way. In E2, accuracy increases modestly, peaking around *r* = 75% at 0.734 and slightly decreasing at *r* = 100% to 0.732. Since GitHub code does not provide legal authority, this gain is unlikely to come from additional legal content. A more plausible interpretation is that generic code contributes implementation scaffolding, such as parsing utilities, branching skeletons, and defensive string handling. These patterns can make the generated program more stable, while the legal conditions themselves still come from *R*_1_ and *R*_2_.

#### 4.3.3 GitHub-only retrieval

The GitHub-only setting clarifies the limit of implementation scaffolding. E3 reaches 0.678, which is above the no-retrieval CoC point of 0.664 but below the full in-domain setting. This shows that generic code priors can help codification even without legal content, but they cannot replace retrieval from legal sources. Together, E1–E3 support a two-layer view of external knowledge for explanation: legal authority and in-domain examples primarily determine *what* conditions should be checked. At the same time, generic code priors affect *how* reliably those checks are implemented.

### 4.4 Source support and explanation failure diagnosis

The previous results show that retrieval-augmented codification improves label accuracy and produces executable explanations. This subsection asks a different question: whether the retrieved legal sources support the conditions used by the explanation trace. A program can run and still apply a condition that is not supported by the retrieved material. Conversely, a textual rationale can mention a relevant rule without making it part of the actual decision procedure. We therefore evaluate predicate support as a check between the conditions used by the explanation and the retrieved legal-source corpus *R*_1_.

For this analysis, we use CAIL and CAP. CAIL represents an element-like charge-prediction setting, where labels are closely tied to statutory distinctions. CAP represents an outcome-style setting, where a binary win/lose label may depend on procedural or evidentiary factors beyond the complaint text. This pair allows us to compare a more source-centered label space with a more institutionally mediated outcome label.

We compute predicate support as a *post-hoc* trace analysis. For the full codification setting, candidate predicates are extracted from the final executable trace, including conditional guards and explicit decision signals. For IRCoT, we extract the corresponding decision conditions from the generated reasoning text. Each candidate condition is then checked against retrieved passages from *R*_1_ by an LLM verifier used as a source-support audit. A condition is counted as supported only when an explicit supporting statement can be matched in the retrieved legal-source corpus; otherwise, it is counted as unsupported. Predicate support is therefore a source-grounding diagnostic. It measures whether the trace uses conditions that are textually grounded in the available retrieval setting; it does not establish legal correctness, doctrinal completeness, or whether the predicate should be dispositive for the outcome.

Table 4 shows that the full codification setting yields higher predicate support than IRCoT on both datasets. On CAIL, supported predicates increase from 97.88% for IRCoT to 99.12% for the full codification setting, and unsupported predicates decrease from 2.12% to 0.88%. The difference is smaller because CAIL is already close to an element-like statutory classification task, where relevant legal sources are often easier to align with explicit conditions. Even there, codification slightly improves the connection between retrieved sources and the applied predicates.

**Table 4:** Explanation-level probes on CAIL and CAP.

Dataset	Method	Supported predicates ↑	Unsupported predicates ↓	CF label correct ↑
CAIL	CoC	–	–	78%
	IRCoT	97.88%	2.12%	84%
	Ours (full)	**99.12%**	**0.88%**	**96%**
CAP	CoC	–	–	44%
	IRCoT	82.63%	17.37%	52%
	Ours (full)	**91.13%**	**8.87%**	**82%**

Predicate support is evaluated against the retrieved legal-source corpus *R*_1_ on 1,000 sampled instances. CoC does not use *R*_1_ during inference and is therefore omitted from the support columns. Counterfactual (CF) label correctness is evaluated on 50 manually edited instances and discussed in Section 4.5. Bold values indicate the best-performing result for the corresponding dataset and metric among the compared methods.

The difference is larger on CAP. Supported predicates increase from 82.63% for IRCoT to 91.13% for the full codification setting, while unsupported predicates decrease from 17.37% to 8.87%. This result is important because CAP has a deceptively simple binary label. The win/lose outcome may involve pleading sufficiency, procedural posture, defenses, or evidentiary state. In such settings, retrieved text can be relevant but still difficult to turn into a correct decision condition. Codification helps by forcing the model to express the retrieved rule as a predicate or branch, making unsupported conditions easier to detect.

The failure diagnosis from these analyses falls into several recurring categories. The first is retrieval miss: the relevant definition, exception, or threshold is not present in the retrieved legal sources. The second is compilation error: the relevant source is retrieved, but the generated predicate implements it incorrectly. The third is unsupported predicate: the trace uses a condition that cannot be backed by *R*_1_. The fourth is label–concept mismatch, where the trace applies a reasonable legal condition but maps it to the wrong benchmark label. The fifth is underdetermination from the record, especially in outcome-style tasks, where the textual input does not contain the procedural or evidentiary information needed to determine the benchmark outcome.

These categories separate different remedies. Retrieval misses suggest improving *R*_1_ coverage or retrieval. Compilation errors suggest improving codification and refinement. Unsupported predicates suggest stronger source-support checking. Label–concept mismatch suggests better label normalization or clearer mapping between legal concepts and benchmark labels. Underdetermination suggests a limitation of the benchmark specification itself rather than a local model failure.

### 4.5 Material-edit sensitivity

We further test whether predictions respond to legally material edits. This probe is applied uniformly to all compared methods, regardless of whether the method outputs text or code. The goal is to test label-level sensitivity: when the input changes in a legally meaningful way, does the predicted label change, or remain invariant, in the expected direction?

For each audited dataset, we sample 50 instances and manually construct counterfactual edits. The edits are designed to preserve the general legal setting while changing a fact that should affect the applicable rule. Examples include flipping an exception, crossing a legally relevant threshold, adding or removing consent, changing a required element, or modifying a temporal condition. We then rerun each method on the edited input and compare the new prediction with the expected counterfactual label.

The last column of Table 4 reports counterfactual label correctness. On CAIL, the full codification setting reaches 96%, compared with 84% for IRCoT and 78% for CoC. On CAP, the full codification setting reaches 82%, compared with 52% for IRCoT and 44% for CoC. These results indicate that retrieval-augmented codification is more sensitive to legally material changes than one-pass codification or interleaved retrieval with textual reasoning.

The difference between CAIL and CAP is also informative. CAIL edits often target statutory elements or charge distinctions, which are relatively direct to express as label-relevant conditions. CAP edits are harder because win/lose outcomes can depend on procedural posture, evidentiary sufficiency, class-certification issues, defenses, and other factors not always recoverable from the complaint text alone. This helps explain why counterfactual label correctness is lower on CAP for all methods.

This probe is intentionally limited. It does not test robustness to arbitrary paraphrases, adversarial perturbations, or full legal counterfactual reasoning. It tests a narrower property: whether a method's final prediction responds correctly to manually controlled, legally material edits. We use it as an explanation-level supplement to accuracy and source support, not as a proof of legal robustness.

## 5 Case studies

We report three qualitative case studies to illustrate how executable explanations can be inspected. For each case, we examine the input, retrieved legal sources when available, the program-level conditions compiled into the trace, and the branch that produces the prediction. The goal is not to show that the full legal decision has been formalized. The goal is to show what kind of explanation the trace provides: which part of the text-to-label path was made explicit, which source-supported conditions affected the output, and where the explanation breaks down.

### 5.1 Success: filling a missing statutory condition in CAP

We first consider a CAP example involving a debt-collection complaint. The input describes an original creditor calling under a false name outside the allowed time window. A one-pass program misses a condition relevant to whether the complaint states a viable claim and predicts the wrong binary outcome. After retrieval, the executable trace adds two missing components: the prerequisite for collector status and the false-name exception. Once these conditions are added, the executed branch changes and the system outputs the correct outcome label.

This case illustrates a useful form of trace-based audit. A small conjunction of explicit conditions produces the final prediction. The relevant legal concepts are visible in the retrieved legal sources, and the branch that changes the output can be inspected. The trace therefore exposes not only the answer, but also the missing condition that caused the initial failure.

### 5.2 Failure A: label-interface error in CAIL

The second case concerns a defendant who resold imported anti-cancer drugs that lacked the required registration in China, profited from multiple sales, and obtained RMB 41,400. The gold label is manufacturing or selling counterfeit drugs, while the predicted label is manufacturing or selling substandard products.

The retrieval returns drug-related criminal provisions from *R*_1_, matching the facts about unregistered imported drugs and resale. The trace also activates drug-related predicates. The failure is therefore not caused by missing legal sources or a non-executable program. Instead, the error occurs at the predicate-to-label interface: the explanation identifies the relevant legal area, but the schema-level mapping selects the wrong benchmark label.

**Simplified explanation trace**.


has_unregistered_import = True
has_resale_for_profit = True

**def** normalize_label(x):
   alias = {"counterfeit_drug":
        "substandard_products"}
   **return** alias.get(x, x)

intended = "counterfeit_drugs"
final_pred = normalize_label(intended)

This case separates two layers of the explanation. The fact-to-predicate layer points to the right legal area, while the predicate-to-label layer fails. This distinction is difficult to obtain from label-only evaluation or from a free-form rationale, but it is visible in the trace.

### 5.3 Failure B: outcome underdetermination in CAP

The third case shows a different boundary. The complaint alleges secret recording of patients in an operating-room setting while they were sedated, without consent or disclosure, and includes class allegations. The gold label is lose, while the executable explanation predicts win.

The trace compiles a complaint-level proxy. It checks for cues such as secret recording, lack of consent, a medical setting, sedation, and class allegations. These cues can support a narrower explanation: the complaint appears to allege conduct consistent with a plausible legal theory. However, a win/lose outcome may also depend on procedural posture, evidentiary sufficiency, standing, damages, class certification, defenses, or judge-specific rulings. These determinants may not be recoverable from the complaint text alone.

**Simplified explanation trace**.


win_score = 0
**if** secret_recording: win_score += 2
**if** no_consent: win_score += 2
**if** medical_setting: win_score += 1
**if** sedated_or_unconscious: win_score += 1
**if** class_allegations: win_score += 1

final_pred = "win" **if** win_score >= lose_score + 2
      **else** "lose"

This case shows a boundary of executable explanation. Even a clean trace may explain only part of the legal decision process. The trace is still useful because it shows which cues drove the prediction and which missing determinants would be needed to justify the benchmark label. In this sense, the failure is not merely a wrong prediction; it is evidence that the benchmark outcome depends on information outside the recoverable record.

## 6 Discussion

### 6.1 Why legal prediction is a difficult setting for XAI

The results show why legal prediction is a difficult setting for explainable AI. A legal benchmark label is rarely a simple surface category. It may compress statutory elements, doctrinal requirements, factual characterization, evidentiary assumptions, procedural posture, and institutional judgment. Direct label prediction hides how these components interact, while a natural-language rationale may describe a plausible legal story without showing whether the relevant condition actually affected the output.

This difficulty varies across legal domains. Some tasks contain relatively rule-like components: definitions, thresholds, temporal requirements, exceptions, and statutory elements. Other tasks require information that is only partially observable from the input record, such as pleading sufficiency, evidentiary support, class-certification issues, defenses, or discretionary judicial assessment. In the latter cases, even a readable explanation may be incomplete because the benchmark label depends on determinants that are not fully present in the input text.

This is why legal prediction is useful as a testbed for executable explanations. It forces the explanation object to connect three layers: facts in the input, legal sources retrieved from outside the input, and the benchmark label. Failures at any of these layers have different meanings. A missing statute, a miscompiled condition, a schema-level label error, and an underdetermined litigation outcome are all different explanation failures, even if they appear only as wrong labels in ordinary accuracy evaluation.

### 6.2 When executable explanations work

Executable explanations are most useful when a benchmark decision can be decomposed into explicit conditions. These conditions include statutory elements, definitions, thresholds, temporal requirements, exceptions, and closed label mappings. In such cases, the text-to-label path can be represented as a small decision procedure: extract relevant facts, check the applicable legal conditions, and map the satisfied conditions to a label.

This explains the strong results on datasets such as CAIL and JTD. Many instances in these datasets involve label distinctions that can be approximated by explicit predicates over facts and retrieved legal sources. For example, a criminal charge may depend on whether a specific act, amount, object, or mental state is present. A tort judgment may depend on whether recognizable liability elements are satisfied. These settings are not trivial, but they provide a relatively clear target for executable explanation because the final label is often connected to identifiable legal conditions.

Executable explanations are less complete when the benchmark label represents a broader institutional outcome. In such cases, the trace may still compute a meaningful legal subroutine, but that subroutine may not be sufficient to recover the benchmark label. This is not merely a model error; it reflects a mismatch between what is recoverable from the input record and what the label operationalizes.

### 6.3 Differences across jurisdictions, languages, and legal domains

A distinctive feature of our evaluation is that it spans several jurisdictions, languages, and legal domains. The five datasets do not only differ in size or label space. They also differ in the kind of legal materials that can support the prediction. CAIL concerns Chinese criminal law, ECHR concerns European human-rights articles, CAP concerns U.S. class-action complaints, ILDC concerns Indian court judgments, and JTD concerns Japanese tort cases. This makes the evaluation useful for studying where executable explanation depends on relatively explicit legal rules and where it encounters broader institutional decision processes.

The cross-dataset results suggest that executable explanations are more stable when the benchmark label is close to identifiable legal elements. CAIL and JTD show high accuracy and high executability. In these settings, many labels can be connected to explicit conditions such as acts, objects, amounts, intent, injury, negligence, or liability elements. The explanation trace can therefore represent a substantial part of the text-to-label path as predicates and branches. This does not mean that criminal or tort reasoning is simple, but it means that the benchmark labels often have a clearer connection to condition-like legal factors.

ECHR and CAP show different difficulties. ECHR requires deeper refinement, suggesting that article prediction often involves reconciling several doctrinal conditions across the case description. CAP has lower executability and a higher exception rate, even though its label is binary. This reflects the fact that U.S. class-action outcomes may depend on procedural posture, pleading sufficiency, certification issues, defenses, and evidentiary context. A binary label can therefore be less explainable through executable conditions than a larger label space if the outcome depends on information that is not explicit in the input record.

The comparison also shows that language is not the only source of variation. The datasets include Chinese, English, and Japanese legal materials, but the main difficulty is not simply multilingual processing. More important is the relation between the input text, the available legal sources, and the benchmark label. A non-English dataset with labels tied to explicit legal elements may be more explainable through executable conditions than an English dataset whose label compresses procedure and litigation outcome. This supports our main claim: retrieval-augmented codification measures not only model performance, but also how much of a legal benchmark decision can be represented as explicit, source-supported conditions.

The kind of law also matters. Criminal-law and tort-law benchmarks often contain elements that can be expressed as fact predicates, even when legal interpretation remains necessary. Human-rights and class-action benchmarks more often require balancing, procedural context, or institutional judgment. Court-outcome datasets may combine all of these factors. These differences caution against treating “legal judgment prediction” as a single homogeneous task. A high or low score can reflect not only model ability, but also the degree to which the benchmark label is recoverable from the input text and retrieved legal sources.

### 6.4 What external knowledge contributes to explanation

The external-knowledge analysis separates legal content from implementation support. Legal-source corpora *R*_1_ provide the conditions to be checked: definitions, elements, thresholds, exceptions, and jurisdiction-specific rules. In-domain examples *R*_2_ provide benchmark-specific mappings between fact patterns and labels. Code-like exemplars and generic code priors mainly support implementation: they help the model express conditions as branches, parsers, and schema-compliant outputs.

These mechanisms should not be conflated. An explanation may improve because the system retrieves the right legal rule. After all, it retrieves a useful solved example, or because it retrieves a code-like structure that stabilizes the generated program. For legal XAI, the important question is therefore not simply whether retrieval helps, but what kind of knowledge is retrieved and how it enters the explanation. Retrieved legal sources affect *what* conditions can be justified, while code-like exemplars affect *how* those conditions are operationalized.

This distinction also clarifies why generic code can help but cannot replace legal sources. GitHub-style code may provide useful implementation patterns, but it does not supply statutory definitions, doctrinal conditions, or jurisdiction-specific exceptions. An executable explanation without legal-source support may still be runnable, but it is not a legally informative explanation.

### 6.5 What explanation traces reveal

Explanation traces provide information that label accuracy does not. A final prediction only tells us whether the output matches the benchmark label. A trace shows how the prediction was produced: what was retrieved, which facts were extracted, which predicates were defined, which branches were taken, and whether the final program *c*^⋆^ executed successfully.

This makes several failure modes distinguishable. A retrieval miss occurs when the required legal source is absent from the retrieved material. A compilation error occurs when the source is retrieved but translated into the wrong predicate or branch. An unsupported predicate occurs when the program uses a condition that cannot be backed by retrieved legal sources. A label-interface error occurs when the trace identifies the right legal area but maps it to the wrong benchmark label. Outcome underdetermination occurs when the input text does not contain enough information to justify the benchmark outcome.

These distinctions are difficult to recover from textual rationales alone. A rationale can mention a relevant rule without actually applying it, and a generated answer can cite legal sources without making clear how those sources affected the prediction. An explanation trace is more restrictive: for a legal condition to affect the output, it must appear as a signal, predicate, or branch. This does not make the result automatically correct, but it makes the decision path more inspectable and the failure modes more diagnosable.

### 6.6 What executability does not establish

Executability is an observability condition, not a correctness guarantee. A running *c*^⋆^ shows that the prediction was produced through an operational procedure, but it does not show that the procedure is legally complete. The program may omit an exception, rely on an unsupported predicate, or compute a proxy that does not match the benchmark label.

The same caution applies to source support and material edits. A source-supported predicate has textual support in the retrieved legal sources, but the complete legal decision may require additional facts, procedures, rule interactions, or discretionary judgment. The material-edit probe tests selected sensitivities, not general legal robustness. These measures are therefore explanation diagnostics, not certificates of legal validity.

This distinction is central to the study's XAI framing. Retrieval-augmented codification does not turn an LLM into a legally authoritative decision-maker. It turns part of the prediction process into an inspectable object. The value of this object is that it makes explicit what was checked, what was supported, and where the explanation fails.

### 6.7 Implications for legal XAI evaluation

The main implication is that evaluation of legal LLMs should not stop at label accuracy or free-form rationales. Accuracy remains necessary because benchmark prediction is still the task being evaluated. But for legal applications, it is also important to know whether the prediction is connected to retrieved legal sources, whether the relevant conditions are explicit, and whether failures can be localized.

Executable explanation traces offer one practical way to add this layer of evaluation. They make it possible to ask more specific questions: Did the model retrieve the relevant legal source? Did it compile the source into the correct predicate? Did the final label depend on that predicate? Did the output fail because of missing retrieval, wrong compilation, label mismatch, or underdetermination from the record?

This view also clarifies the role of legal benchmarks. Some benchmarks are well suited to executable explanation because their labels correspond to relatively explicit legal conditions. Others are useful precisely because they expose the boundary of explanation: the model can construct a plausible legal subroutine, but the benchmark outcome depends on information outside the input text. Treating these failures as diagnostic rather than merely negative can make legal XAI evaluation more informative.

In this sense, retrieval-augmented codification changes the role of failure. When a trace succeeds, it shows which part of the decision path can be made explicit. When it fails, it helps identify whether the obstacle lies in retrieval, codification, label mapping, or information absent from the record.

### 6.8 Limitations

This study has several limitations. First, the results depend on the base LLM, decoding settings, prompts, retrieval budget, and refinement cap. We control the base model, decoding settings, retrieval configuration, and context budget whenever applicable, but refinement changes the inference process for iterative variants. Different models may generate different programs, retrieval queries, and schemas. The reported numbers should therefore be read as empirical results under the specified runtime setting, not as model-independent properties of executable legal traces.

Second, the quality of the trace depends on the construction of *R*_1_ and *R*_2_. The legal-source corpus *R*_1_ may be incomplete, unevenly chunked, outdated, or insufficient for questions requiring procedure, evidence, or case-specific precedent. The in-domain example pool *R*_2_ is built fold-safely to avoid direct test leakage, but it can still encode benchmark-specific mappings. Improvements from *R*_2_ should therefore be interpreted as task-specific support, not necessarily as transferable legal reasoning. These issues are amplified in cross-jurisdictional evaluation. The five benchmarks differ not only in language and legal domain, but also in legal culture, procedural assumptions, label construction, and the availability of external legal sources. Our cross-jurisdictional setting is therefore intended to test the robustness of the trace-based audit framework across heterogeneous legal prediction tasks, not to claim a jurisdiction-independent legal reasoning model.

Third, legal benchmark labels compress complex decision processes. Some labels correspond closely to statutory elements or article applicability, while others depend on evidence, procedure, judicial discretion, or institutional practice. When such determinants are absent from the input text, an executable trace cannot recover them from the record alone. A running *c*^⋆^ makes the prediction operationally inspectable, but it does not imply that the program is legally complete, normatively valid, or sufficient for decision support.

Finally, this study does not include human evaluation of explanation quality. Conventional XAI studies often evaluate explanations through human ratings of clarity, usefulness, or trustworthiness. Such evaluations are appropriate when explanations are intended primarily for human consumption. In contrast, the codified traces produced in our framework are executable artifacts designed for formal inspection, verification, and error diagnosis. Their primary consumers are not end users, but analysts, auditors, and downstream computational processes. We therefore evaluate them through executability, source support, and sensitivity to evidence modifications rather than subjective human judgments. This does not remove the need for future human-facing evaluation; it clarifies the scope of the present study. We study auditability as a step toward explainability, not as a replacement for human-facing legal explanation.

## 7 Conclusion

The technical basis of this study is retrieval-augmented codification as an auditability-oriented approach to legal LLM prediction. The central question was not whether legal decision-making can be fully reduced to code, nor whether generated programs constitute complete human-facing explanations. Rather, we asked whether direct LLM outcome generation can be turned into an executable trace that exposes parts of the text-to-label path for computational inspection.

Across five legal benchmarks, retrieval-augmented codification produced executable traces while preserving predictive utility. The full codification setting improved mean label accuracy over prompting, retrieval, and one-pass codification baselines. More importantly, the traces made the decision process more inspectable: they showed which materials were retrieved, which conditions were compiled into predicates and branches, whether the final program executed, and where the procedure failed. This allowed us to distinguish failures that are otherwise hidden by label accuracy, including missing legal sources, incorrect compilation, unsupported predicates, label-interface errors, and outcome underdetermination.

The results clarify the role of executable traces in legal XAI. They are most informative when benchmark decisions depend on definitions, thresholds, exceptions, statutory elements, or other rule-like components. They are less complete when labels compress procedural posture, evidentiary assumptions, institutional judgment, or discretionary factors outside the input record. In these cases, the trace may still identify a useful legal subroutine, but it also reveals why the benchmark label cannot be fully explained from the available record.

The main value of retrieval-augmented codification is therefore diagnostic. A runnable program does not establish legal correctness, and a source-supported predicate does not amount to a complete legal decision. However, executable traces provide a practical way to inspect how legal knowledge enters a prediction, which conditions affect the output, and where the reasoning process breaks down. This positions retrieval-augmented codification as a tool for auditable legal AI and as a step toward more robust explainable legal LLM systems.

## Data Availability

The original contributions presented in the study are included in the article/Supplementary material, further inquiries can be directed to the corresponding author.
