# Supplementary material for: Executable explanation traces for legal LLM predictions via retrieval-augmented codification
Source: Front Artif Intell. 2026 Aug 20;9:1905145. doi: 10.3389/frai.2026.1905145 (PMC13538117; doi:10.3389/frai.2026.1905145)
Supplement: Supplementary file 1 [file Supplementary_file_1.pdf]

Supplementary Materials

Haoyang Chen [1,*] **and Kumiko Tanaka-Ishii** [1]

[1] *Department of Computer Science and Communications Engineering, School of Fundamental Science and Engineering, Waseda University, Tokyo, Japan*

Correspondence*:
Haoyang Chen
haoyangc0308@fuji.waseda.jp

1 IMPLEMENTATION DETAILS

1.1 Building the Code Exemplar Pool

The in-domain example corpus R_2 is constructed offline before evaluation. Its purpose is to provide fold-safe solved examples that can be retrieved as analogical support during codification. We use two forms of R_2: natural-language solved examples and code-like exemplars.

Step 1: solved example to code-like snippet. For the code-like version of R_2, each solved training instance is translated into a short Python-like snippet using a fixed conversion prompt. The snippet represents the mapping from legally relevant facts to the benchmark label. The conversion is performed only for training instances available in the corresponding fold; held-out test instances are excluded from R_2.

Step 2: indexing. The resulting examples are indexed for retrieval. Each indexed item stores the original example identifier, the textual form, the code-like snippet when available, and the associated label. At test time, the retriever returns the top-k nearest examples from the fold-safe pool. The retrieved examples are then provided either as natural-language examples or as code-like exemplars, depending on the method variant.

Leakage control. For each evaluation fold, R_2 is rebuilt from the training portion only. The held-out instances are never indexed as solved examples for their own evaluation fold. This prevents direct answer lookup while preserving the intended case-based retrieval setting.

1.2 Shared Runtime Settings

Table 1 reports the run-time settings shared across the experiments.

LLM settings. We use the chat-completion interface with a single system message and the temperature listed in Table 1. The base model, decoding settings, retrieval top-k, and context budget are fixed whenever applicable. For codified variants with refinement, the loop is capped at $N_{\max} = 10$. Because refinement changes the inference budget relative to one-pass methods, we report the observed refinement depth N and include $\text{Ours}_{\text{1PASS}}$ as a no-refinement ablation.

Retrieval settings. All retrieval-based methods use the same retrieval top-k and the same fold-safe R_2 construction. The difference between RAG_{NL}, RAG_{CODE}, the text-example codification variant, and the full codification setting lies in how the retrieved items are represented and whether they are compiled into a codified decision procedure.

Table 1. Run-time settings shared across all experiments.

(a) Inference hyper-parameters

Parameter	Symbol	Value
Main LLM backend	–	GPT-5
Code-exemplar backend	–	GPT-5
Model temperature	τ	0.2
Maximum refinement rounds	$N_{\max}$	10
Evaluation folds	k	5
Output format	–	JSON prediction + signals

(b) Retrieval-index configuration

Setting	Value
Embedding model	all-MiniLM-L6-v2
Embedding dimension	384
Index type	FAISS Flat L2
Retrieval top-k	5
Mean code-like snippet length	$\approx$ 18 LOC
Corpus rebuilds	Once offline per dataset and fold

2 PROMPT TEMPLATES

This appendix reports the three prompt templates used in the retrieval-augmented codification pipeline: the initial codification prompt, the iterative revision prompt, and the R_2 snippet-generation prompt. Dataset-specific label lists, retrieved passages, and input texts are inserted into the corresponding placeholders at run time.

2.1 Initial Codification Prompt

You are given a legal benchmark instance described in natural language.

Your task is to translate the input into an executable Python program.

The program should be executable as a standalone solution for the given instance. It should make important facts, legal conditions, exceptions, and label decisions explicit in code.

The program should solve the instance by an executable procedure rather than by free-form explanation. Important reasoning steps should be expressed through executable statements rather than comments alone.

The program must output the final prediction in the required machine-readable schema.

[Benchmark input t, task description, and allowed label schema inserted here.]

Return only the Python program.

2.2 Iterative Revision Prompt

You are revising an executable Python program for a legal benchmark instance.

Given the benchmark input, the current program, retrieved legal authority, retrieved examples, and feedback from the previous attempt, produce:

(i) a revised Python program, and

(ii) a retrieval query for missing information, if additional legal authority or examples are needed.

The revised program should preserve correct parts where possible. It should make supported facts, legal conditions, procedures, exceptions, and constraints more explicit in code.

If the retrieved legal sources supports a condition, encode the condition as an executable predicate or branch. If a required condition is missing, issue a concise retrieval query.

[Benchmark input, current code, retrieved legal sources R_1, retrieved examples R_2, and feedback inserted here.]

Output format:

[REVISED_PROGRAM]

...

[RETRIEVAL_QUERY]

...

2.3 R_2 Snippet-Generation Prompt

You are given a solved legal benchmark example.

Your task is to produce a short Python snippet that captures a useful condition, check, or procedure from the example in executable form.

The snippet should provide structural guidance for solving similar benchmark instances.

Requirements:

- Use Python.
- Keep the snippet short and focused.
- Make the relevant condition, check, or procedure explicit in code.
- Prefer reusable executable structure over benchmark-specific surface wording.
- Do not include free-form explanation outside the code.

[Solved benchmark example and gold label inserted here.]

Return only the Python snippet.

3 PREDICATE-SUPPORT VERIFICATION PROCEDURE

Predicate support is evaluated by an LLM verifier as a post-hoc source-grounding audit. For each instance, we provide the verifier with the extracted candidate predicate, the predicted label, and the retrieved R_1 passages available to the model. The verifier is instructed to mark a predicate as supported only when an explicit statement in the retrieved legal-source passages supports the condition used by the trace. If the support is implicit, absent, contradicted, or only inferable from unstated assumptions, the predicate is marked as unsupported. The verifier output is restricted to a binary support label and a short source pointer or justification.

These labels are used only as diagnostic estimates of source grounding. They are not treated as human legal annotations, doctrinal correctness judgments, or evidence that the predicate is sufficient for the benchmark label.

4 DATASET AND LEGAL-SOURCE RECONSTRUCTION

Table 2 summarizes the benchmark sources and the legal-source materials used to construct R_1. The benchmark datasets and legal texts are not redistributed by this paper when they are governed by third-party licenses, access conditions, or official distribution channels. Instead, users should obtain the corresponding materials from the original providers and apply the preprocessing, chunking, indexing, and retrieval

Table 2. Benchmark and legal-source reconstruction pointers. Benchmark datasets and legal-source corpora should be obtained from their original providers subject to their respective licenses and access conditions.

Dataset	Benchmark source	R_1 legal-source material	Reconstruction note
CAIL	CAIL2018 benchmark and competition source Xiao et al. (2018); official competition site: `http://cail.cipsc.org.cn/`	Chinese Criminal Law and related judicial interpretations used for charge prediction.	Obtain the benchmark from the official CAIL distribution. Construct R_1 from the corresponding criminal-law provisions and related interpretive materials, then apply the chunking and retrieval settings described in Section 1 of this Supplementary Material.
ECHR	ECHR legal judgment prediction dataset Chalkidis et al. (2019)	European Convention on Human Rights articles and related legal materials from official ECHR/HUDOC sources.	Obtain the benchmark from the original dataset provider. Construct R_1 from the Convention articles and official legal-source materials used in the experiment.
CAP	ClassActionPrediction benchmark Semo et al. (2022); dataset sample: `https://huggingface.co/datasets/darrow-ai/USClassActions`; expert annotations: `https://huggingface.co/datasets/darrow-ai/USClassActionOutcomes_ExpertsAnnotations`; code: `https://github.com/darrow-labs/ClassActionPrediction`	U.S. legal materials relevant to class-action outcome labels, including public legal-source materials used for retrieval.	Obtain the released class-action complaints and labels from the original provider. Construct R_1 from the public U.S. legal materials described in the dataset and preprocessing notes.
ILDC	Indian Legal Documents Corpus for Court Judgment Prediction and Explanation Malik et al. (2021)	Indian legal materials relevant to Supreme Court judgment prediction.	Obtain the benchmark from the original ILDC provider. Construct R_1 from the public legal-source materials used in the experiment and index them using the same retrieval settings.
JTD	Japanese Tort-case Dataset for rationale-supported legal judgment prediction Yamada et al. (2025)	Japanese Civil Code and related legal materials used for tort judgment prediction.	Obtain the benchmark from the original JTD provider. Construct R_1 from the Japanese Civil Code and related legal-source materials, then apply the preprocessing and indexing settings described in Section 1 of this Supplementary Material.

settings described in Appendix 1. This reconstruction procedure is intended to make the evaluation pipeline reproducible without redistributing materials whose distribution is not controlled by the authors.

REFERENCES

Chalkidis, I., Androutsopoulos, I., and Aletras, N. (2019). Neural legal judgment prediction in English. In *Proceedings of the 57th Annual Meeting of the Association for Computational Linguistics* (Florence, Italy: Association for Computational Linguistics), 4317–4323. doi:10.18653/v1/P19-1424

Malik, V., Sanjay, R., Nigam, S. K., Ghosh, K., Guha, S. K., Bhattacharya, A., et al. (2021). ILDC for CJPE: Indian legal documents corpus for court judgment prediction and explanation. In *Proceedings of the 59th Annual Meeting of the Association for Computational Linguistics and the 11th International Joint Conference on Natural Language Processing (Volume 1: Long Papers)* (Online: Association for Computational Linguistics), 4046–4062. doi:10.18653/v1/2021.acl-long.313

Semo, G., Bernsohn, D., Hagag, B., Hayat, G., and Niklaus, J. (2022). ClassActionPrediction: A challenging benchmark for legal judgment prediction of class action cases in the US. In *Proceedings of the Natural Legal Language Processing Workshop 2022* (Abu Dhabi, United Arab Emirates (Hybrid): Association for Computational Linguistics), 31–46. doi:10.18653/v1/2022.nllp-1.3

[Dataset] Xiao, C., Zhong, H., Guo, Z., Tu, C., Liu, Z., Sun, M., et al. (2018). CAIL2018: A large-scale legal dataset for judgment prediction

Yamada, H., Tokunaga, T., Ohara, R., Tokutsu, A., Takeshita, K., and Sumida, M. (2025). Japanese tort-case dataset for rationale-supported legal judgment prediction. *Artificial Intelligence and Law* doi:10.1007/s10506-024-09402-0
